# Investigating the role of stable flies (*Stomoxys calcitrans*) and biting midges of the genus *Culicoides* as potential mechanical vectors of African swine fever virus in Lithuania, Poland and Romania

**DOI:** 10.1186/s13071-025-06816-w

**Published:** 2025-07-31

**Authors:** Sofie Dhollander, Oana-Maria Balmoș, Eleonora Cattaneo, José Abrahantes Cortiñas, Anette Ella Boklund, Anna Szczotka-Bochniarz, Andrei Daniel Mihalca, Lina Mur, Maciej Frant, Anna Gal-Cisoń, Malgorzata Kwasnik, Wojciech Rozek, Alvydas Malakauskas, Marius Masiulis, Jurga Turcinaviciene, Theodora Chesnoiu, Krzysztof Jazdzewski, Jerzy Rola, Florica Barbuceanu, Miguel Ángel Miranda, Jan Arend Stegeman

**Affiliations:** 1 Assess/Enable Department, European Food Safety Authority, Parma, Italy; 2 Department of Veterinary and Animal Sciences, University of Copenhagen, Copenhagen, Denmark; 3 Department of Cattle and Sheep Diseases, National Veterinary Research Institute, Pulawy, Poland; 4 Department of Parasitology and Parasitic Diseases, University of Agricultural Sciences and Veterinary Medicine of Cluj-Napoca, Cluj-Napoca, Romania; 5 Department of Swine Diseases, National Veterinary Research Institute, Pulawy, Poland; 6 Department of Virology, National Veterinary Research Institute, Pulawy, Poland; 7 Veterinary Academy, Lithuanian University of Health Sciences, Kaunas, Lithuania; 8 Department of Zoology, Institute of Biosciences, Life Sciences Centre of Vilnius University, Vilnius, Lithuania; 9 Department of Animal Health and Welfare, National Sanitary Veterinary and Food Safety Authority, Bucharest, Romania; 10 Chief Veterinary Office, General Veterinary Inspectorate, Warsaw, Poland; 11 Pathology Department, Institute for Diagnosis and Animal Health, Bucharest, Romania; 12 Department of Clinical Sciences 2, University of Agronomical Sciences and Veterinary Medicine of Bucharest, Bucharest, Romania; 13 Department of Biology, ZAP-UIB, INAGEA-UIB, University of the Balearic Islands, Palma, Balearic Islands Spain; 14 Department of Farm Animal Health, Utrecht University, Utrecht, The Netherlands

**Keywords:** *Stomoxys calcitrans*, Culicoides, Mechanical transmission, African swine fever virus, Pigs

## Abstract

**Background:**

Since its emergence in Georgia in 2007, the seasonal pattern of African swine fever virus (ASFV) genotype II outbreaks in European pig populations has been evident. It is hypothesized that summer-related farming practices, along with the increased activity and abundance of arthropod vectors during warmer months, contribute to the increased incidence of these outbreaks during this period. This study investigated the potential role of stable flies (*Stomoxys calcitrans*) and biting midges of the genus *Culicoides* as mechanical vectors of ASFV. In addition, the potential distribution and abundance of different species of *Culicoides* biting midges on pig farms was investigated.

**Methods:**

From August 2021 to August 2023, vector surveillance was conducted as part of a case–control study on 42 outbreak farms and 70 control farms across Romania, Lithuania, and Poland. Collected insect specimens were pooled and tested for ASFV DNA using real-time polymerase chain reaction (PCR).

**Results:**

A total of 8604 biting midges of the genus *Culicoides* specimens and 742 *S. calcitrans* flies were collected, with ASF DNA detected in 27 out of 1219 insect pools. Positive pools were predominantly observed in *Culicoides punctatus*, *C. newsteadi*, and the Obsoletus complex, with most detections occurring in August. However, ASFV isolation was unsuccessful. Statistical analyses revealed no significant association between farm status (outbreak versus control) and ASFV DNA detection in pools of biting midges of the genus *Culicoides*, likely due to limited sample size. Additionally, two ASFV-positive *S. calcitrans* pools were identified, supporting their potential role as mechanical vehicles for ASFV. The findings highlight the affinity of *C. punctatus*, *C. newsteadi*, and the Obsoletus complex and *S. calcitrans* for pig farms – an aspect that was previously undocumented. This association may increase the likelihood of ASFV acquisition and dissemination by these species. Environmental factors, such as pig farm density and proximity to other hosts, likely influence this risk.

**Conclusions:**

These results emphasize the importance of vector control strategies, including insect netting, to mitigate ASFV transmission risks. Further research is needed to understand the dynamics of ASFV infection in arthropods. Notably, this study also reports the first identification of *Culicoides riethi* and *Culicoides salinarius* in Lithuania.

**Graphical abstract:**

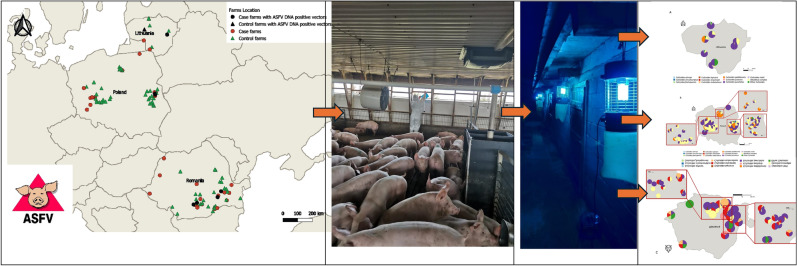

## Background

African swine fever (ASF) is a highly contagious and severe viral disease of domestic and wild suids, caused by a double-stranded DNA virus of the Asfarviridae family, genus *Asfivirus* [1]. The virus spreads through direct contact with infected animals or their secretions, and indirectly via contaminated feed or fomites [2, 3]. During the last 10 years. ASF has caused devastating economic losses globally, affecting both backyard and commercial pig farmers. The disease has disrupted pork supply chains, led to the culling of millions of pigs, and significantly increased pork prices. Overall, the global pork industry has faced losses running into billions of dollars due to ASF outbreaks [4].

In Europe, the epidemiological situation worsened in 2023, with ASF virus (ASFV) genotype II affecting 14 EU Member States, including new outbreaks in Croatia and a resurgence in Romania. The number of outbreaks among domestic pigs in 2023 was five times higher than in 2022, causing substantial disruptions to the EU’s pork production and trade. ASF outbreaks in domestic pigs were clearly seasonal in all countries, with 88% of outbreaks reported between July and October [5]. The seasonal nature of ASFV genotype II outbreaks in pigs in Europe has been evident since its introduction in Georgia in 2007. Summer-related farming practices, such as feeding freshly cut forages and growing of attractive crops for wild boar near pig farms, have been hypothesized to contribute to the increased risk of virus introduction on farms. Additionally, the transmission by arthropod vectors, which are more abundant and active during the warmer months, has also been suggested as a contributing factor [6–12].

Species of soft ticks from the *Ornithodoros erraticus* complex (Ixodida; Argasidae) is currently the only confirmed biological vector of ASFV transmission in Europe. The biological transmission cycle with pigs has been demonstrated using field strains of ASFV collected during the epidemic in the Iberian Peninsula ([13–15]). However, with the currently circulating Georgia 2007/1 ASFV strain, only oral transmission by feeding pigs with infected ticks has been demonstrated [16], despite infectious virus being detectable in ticks up to 8 months after exposure, no transmission by feeding ticks was observed [17]). Thanks to its capacity to harbour infectious ASFV for several months, and its nidicolous behaviour (i.e. inhabiting burrows or cracks and feeding for short periods), *O. erraticus* is considered a reservoir of ASFV in outdoor farms. In such environments, these ticks can thrive and remain close to pigs. However, they are not considered to play a relevant role in spreading the virus between farms [18].

Furthermore, *O. erraticus* and other potential soft tick vector species have not been observed in most areas currently affected by ASFV Georgia 2007/1 in Europe, making it highly unlikely that they are responsible for the peak in incidence during the summer months in these regions [5]. However, mechanical transmission by arthropod vectors that are abundant during the warmer months has been the focus of several field studies in affected regions. Mechanical transmission occurs when an arthropod passively carries a pathogen on its body (such as its mouthparts, legs, or exoskeleton) without the pathogen undergoing any biological development or replication inside the vector. This differs from biological transmission, in which the pathogen depends on the vector for part of its life cycle [19].

DNA of ASFV genotype II has been detected in biting insects such as biting midges of the genus *Culicoides*, i.e., in *C. obsoletus*, *C. newsteadi*, *C. punctatus*, *C. nubeculosus*, *C. festivipennis*, *C. lupicaris*, *C. pulicaris*, *C. puncticollis*, and *C. submaritimus*[20], mosquitoes (Culicidae) [20, 21] and *Stomoxys calcitrans* [20, 22] near or on outbreak farms. In addition, ASFV DNA-positive hematophagous flies (i.e. Tabanidae) have been identified on or near ASF-free pig farms [9, 22]. Still, to date, no studies have successfully isolated infectious ASFV from these biting insects. The transmission of ASFV to pigs by arthropods feeding on pigs has only been demonstrated under laboratory conditions. For instance, Mellor et al. [23] showed that *S. calcitrans* adults fed on ASFV-infected blood were subsequently able to infect pigs during subsequent feedings on pigs.

Carn [19] reviewed the role of mechanical transmission by various haematophagous species of Diptera, highlighting *Stomoxys calcitrans* as a particularly effective mechanical vector. This species has been shown to successfully transmit lumpy skin disease virus, sheep and goat pox virus and *Trypanosoma evansi* mechanically, attributed to its large mouth parts and its feeding behaviour, which involves taking multiple and large blood meals two to three times per day. Further, *Stomoxys calcitrans* is a strong and active flier, which enhances its potential as a mechanical vector. Baldacchino et al. [24] came to the same conclusions in their review. In contrast, *Culicoides* are less obvious candidates for mechanical transmission. Their bites are less painful, making them less likely to be dislodged by their hosts, and their flight ranges are relatively limited. There is also a lack of knowledge around the impact of partial blood feeding in *Culicoides*, whereas it is very common for *Stomoxys* to take multiple blood meals. Nonetheless, they have been implicated in mechanical transmission of Fowl pox [25] and Rift Valley fever [26]. In addition, their high abundances in certain areas may compensate for their lower transmission efficacy. This study aimed to gather field evidence on the potential role of stable flies and biting midges of the genus *Culicoides* in the mechanical transmission of ASFV. Conducted as part of a case–control study between 4 August 2021 and 23 August 2023 in Romania, Lithuania and Poland [27], the study hypothesized that, if these insect groups contribute to ASFV transmission, the virus would at least be detectable in or on the insects. Such detection would indicate that the insects had fed on infectious blood or secretions, potentially serving as vehicles for introducing infectious virus onto ASF-free farms. Furthermore, a higher proportion of ASFV-positive insects or insect pools on affected farms compared with control farms would support their role in transmission within farms. Conversely, the detection of ASFV-positive insects on control farms could indicate the potential of these insects to transport the virus onto ASFV-free farms.

Additionally, the collected data on biting midge species and *S. calcitrans* were analysed to identify species more likely to carry ASFV DNA and to investigate the presence of new *Culicoides* species in previously unreported areas.

Finally, this study aimed to identify the affinity of *Culicoides* species for pig farms. Few studies have specifically investigated *Culicoides* species feeding on pigs in Europe. This is likely because *Culicoides* research has primarily centred on their role as vectors of pathogens that affect ruminants (e.g. Schmallenberg virus and bluetongue virus) or horses (e.g. African horse sickness virus). A systematic review of *Culicoides* host-feeding patterns indicated that, while most species predominantly feed on ruminants and horses, certain biting midges of the genus *Culicoides*, such as Obsoletus and Pulicaris complexes, exhibit opportunistic feeding behaviours. These species can feed on multiple host species, including pigs, depending on host availability and abundance [28, 29]. Therefore, in this study the aim was to identify presence and abundance of different *Culicoides* species on pig farms.

## Methods

### Site selection

This study was part of a prospective case–control study that took place between 4 August 2021 and 23 August 2023 to investigate potential risk factors for the introduction of ASFV on commercial pig farms in Romania, Lithuania and Poland [27]. For each commercial pig farm where an ASF outbreak was confirmed, two control farms were selected, matched by herd size (e.g. 30–200 pigs, 201–1000 pigs, > 1000 pigs) and by region, i.e., the Nomenclature of Territorial Units for Statistics (NUTS) 3 region in which the farm was located. If no control farm could be selected in the same NUTS 3 region, a control farm from the same herd size category was chosen from elsewhere in the country. Following the confirmation of the ASF outbreaks on commercial pig farms by the official Veterinary Services in Lithuania, Poland and Romania, the entomology teams were immediately informed about the locations of the affected farms to start vector collection. Simultaneously, two control farms were randomly assigned to each case farm by the team in the European Food Safety Authority (EFSA). Case farms were to be visited within 2 days of ASF confirmation, and control farms within 2 weeks after the matching case farm’s outbreak confirmation.

### Vector traps

#### Stable flies (*Stomoxys calcitrans*)

Blue sticky traps were used to collect stable flies, on the basis of the method described by Hogsette and Kline [30]. Four trap locations were selected on each farm, two inside and two outside the pig sheds. The traps were placed at least 15 m apart in areas with high fly activity and direct sunlight to maximize visibility to stable flies. Outdoor traps were placed near the animals, at maximum 15 m from the shed at a height of 1.6 m. Traps were deployed for 1 day, covering the entire daylight period, as stable flies are diurnal and attracted to translucent and blue-coloured objects.

All stable flies were collected in dry containers and kept cool/frozen (+4 °C to −20 °C) during transport. Upon arrival at the laboratory, samples were immediately stored at –80 °C until morphological identification, ASFV DNA testing and blood meal source analysis.

#### Biting midges of the genus *Culicoides*

Centers for Disease Control (CDC) miniature light traps equipped with 4 W ultraviolet (UV) black light tubes were used to collect biting midges of the genus *Culicoides* following the methodology according to Medlock et al. [31]. Collection of bigger insects were avoided by a net filter (mesh size 5 mm) placed between the light source and the suction fan.”

Two traps were placed on each farm: one inside and one outside the pig shed. Traps were installed at a height of 1.5–2 m above the ground, as recommended by Diarra et al. [32]. Outdoor traps were placed in areas protected from wind and rain. Traps were situated in dark locations to minimize competition with other light sources. On the basis of the feeding activity pattern of biting midges of the genus *Culicoides*, feeding mainly from dusk to dawn [33], traps were activated 1 h before sunset and deactivated 1 h after sunrise, facilitating overnight collection of biting midges of the genus *Culicoides* on each farm. The midges were left alive overnight. Samples were collected early in the morning, placed in dry containers, and stored as quickly as possible at −80 °C, allowing identification of *Culicoides* spp., virus isolation and blood meal source identification at a later stage.

### Vector species identification

The identification of the arthropods was based on morphological characteristics. Identification of *S. calcitrans* was performed using descriptions and keys provided by Zumpt [34] and Foil and Hogsette [35]. *Culicoides* spp. were identified using the Interactive Identification Key for *Culicoides* (IIKC), encompassing 110 taxonomic units (102 species and eight morphological variants) as detailed by Mathieu et al. [36]. According to Augot et al. [37], *Culicoides obsoletus*, *Culicoides scoticus*, and *Culicoides chiopterus* were considered to be part of the Obsoletus group based on molecular phylogenetic studies. In the traditional taxonomy, however, the Obsoletus complex is used, comprising the species *C. obsoletus* and *C. scoticus*, and *C. chiopterus* is not part of the Obsoletus complex, as it is distinguishable morphologically and thus the Obsoletus complex was used in this study. Identification of the vector species was carried out in the laboratories of each of the countries from which insects were collected, that is the Vilnius University, Life Sciences Centre in Lithuania; the University of Agricultural Sciences and Veterinary Medicine in Cluj-Napoca in Romania; and the Veterinary Research Institute in Pulawy in Poland. The latter also performed the virus isolation for all three countries involved in the study.

### Detection of ASFV and its DNA and pig DNA in vector samples

#### Pooling of samples

Prior to DNA extraction based on Cêtre-Sossah et al. [38] and Şevik and Oz [39], arthropod samples were pooled by species, collection site and date. Stable flies were pooled in pools of up to maximum four specimens, while biting midges of the genus *Culicoides* were pooled in pools of maximum 20 specimens. Before pooling, each individual stable fly was dissected longitudinally, creating two identical aliquots. For each individual dissection, sterile scalpel blades were used. These aliquots were then homogenized (with pools containing 1–4 halves of stable flies). This approach allowed for obtaining material for both polymerase chain reaction (PCR) and potential subsequent virus isolation while reducing the number of homogenizations.

Biting midges of the genus *Culicoides* were pooled in groups of 1–20 individuals, homogenized and subsequently divided into two equal portions. The first pool of aliquots of stable flies and the first portion of biting midges of the genus *Culicoides* was used for detection of ASFV DNA by real-time PCR. Additionally, in Romania, the first aliquots also underwent PCR analysis to detect pig DNA. The second pool of aliquots was reserved for virus isolation, if the corresponding first pool tested positive by real-time PCR with a cycle threshold (Ct) value below 20.

#### ASFV DNA identification in vectors

All pooled arthropod samples, including stable flies and biting midges of the genus *Culicoides*, were tested for ASFV DNA by real-time PCR according to the standard operational procedures (SOPs) used by the ASF national laboratories in Poland (National Veterinary Research Institute, Pulawy), Romania (Institute for Diagnosis and Animal Health, Bucharest), and Lithuania (Lithuanian University of Health Sciences and the Life Sciences Centre of Vilnius University). These SOPs align with those of the European Union Reference Laboratory for ASF.

The pooled arthropod samples were ground in MagNa Lyser Green Beads with 1.5 mL cold phosphate-buffered saline (PBS 1×) containing 0.1% gentamicin sulphate. Suspensions were clarified by centrifugation at 5000*g* for 5 min, and 200 µL supernatant was used immediately for ASFV DNA detection or store at < −70 °C for future use. Ct values below 35 were considered positive for ASFV DNA. Validated extraction and amplification kits, routinely used for detection of ASFV in animal samples, were used, adhering to the SOPs implemented at the National Reference Laboratory (NRL) for ASF.

#### Detection of pig DNA

Pig DNA analysis was conducted to evaluate whether the vectors had been in contact with pigs or pig secretions (including wild boar or domestic pigs), to check whether the ASFV DNA-positive vectors were exposed to pigs. Only pools positive for ASFV DNA were tested for the presence of pig DNA. All positive samples for ASFV genome were tested by conventional PCR using an in-house method developed by the ASF NRL of the Institute for Diagnosis and Animal Health (IDAH) on basis of the protocol described by the Dalmasso et al. [40] to detect mitochondrial DNA of different mammal species.

#### Virus isolation in vectors

Samples that tested positive upon real-time PCR for ASFV DNA with Ct values lower than 20 were further analysed for virus isolation. Only strong positive pools were considered to be worth the resources to isolate ASFV, as described earlier [41, 42]. The National Veterinary Research Institute in Pulawy, Department of Swine Diseases, performed the virus isolation for the three countries participating in the study. The potential presence of infectious virus in the insects homogenates was assessed using a hemadsorption assay in 96-well plates. An aliquot of 50 µL of each filtrated sample (0.45 µm Sartorius syringe filters, Göttingen, Germany) was added (in triplicate) to primary porcine alveolar macrophage (PPAM) cell culture or similar cultures supporting the growth of ASFV, such as porcine leukocytes or bone marrow. The cultures contained 200 µL of growth medium: Roswell Park Memorial Institute (RPMI) (Gibco, Waltham, USA), 10% of foetal bovine serum (Gibco, Waltham, USA), 1% of antibiotic/antimitotic solution (Sigma Aldrich, St. Louis, USA) and 1:300 (v/v) swine red blood cells (RBC). Hemadsorption was monitored via microscopy over 5 days post-inoculation, as described by Walczak et al. [43].

### Data collection, analysis and visualization

The results of the vector identification and laboratory tests conducted in the three countries were standardized, cleaned and processed using MS Access. Maps were created using R software 4.0. To study whether there was a difference between the presence of ASFV-DNA-positive vector pools in the case and control farms three different analyses were conducted on the *Culicoides* results: random Forest analysis, classification tree analysis and logistic regression analysis. Different variables were investigated for their potential influence on the ASFV DNA test results of the *Culicoides* spp. pools: the sampling year, sampling month, presence of pigs on the farm at the moment of placing the vector traps, numbers of insects in the pool, country, region, farm category (case or control) and the *Culicoides* species. No statistical tests were performed on the *S. calcitrans* results, due to the small numbers of pools investigated.

## Results

### Geographic distribution of *Culicoides* species

**Fig. 1:**
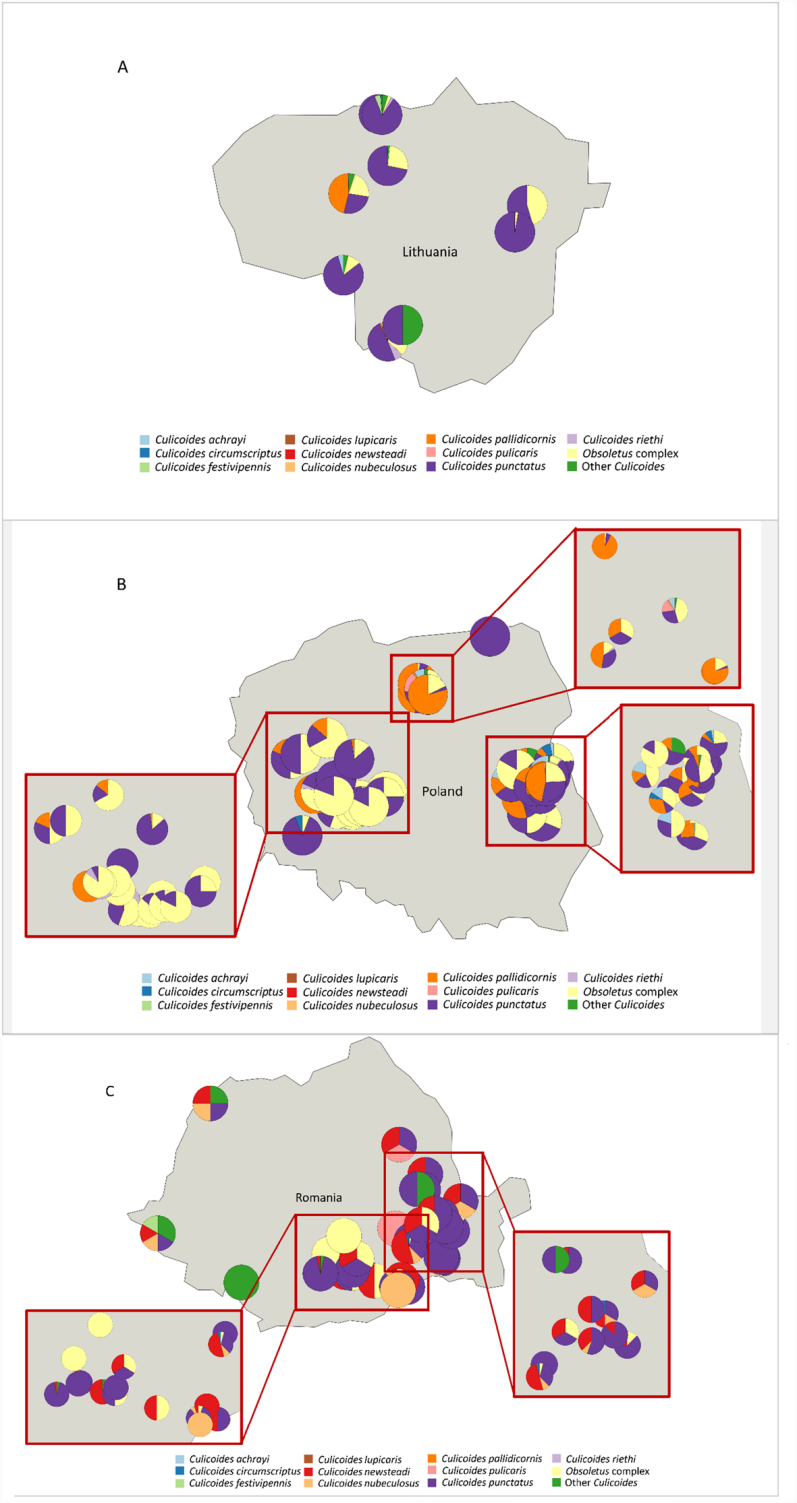
Distribution of *Culicoides* species across farms participating in the vector surveillance in Lithuania (**A**), Poland (**B**) and Romania (**C**)

Figure 1 displays the geographic distribution of the different *Culicoides* species that were observed on the 112 commercial pig farms included in the study, showing the predominance of *C. punctatus,* Obsoletus complex, *C. pallidicornis* and *C. newsteadi* on the pig farms.

**Fig. 2:**
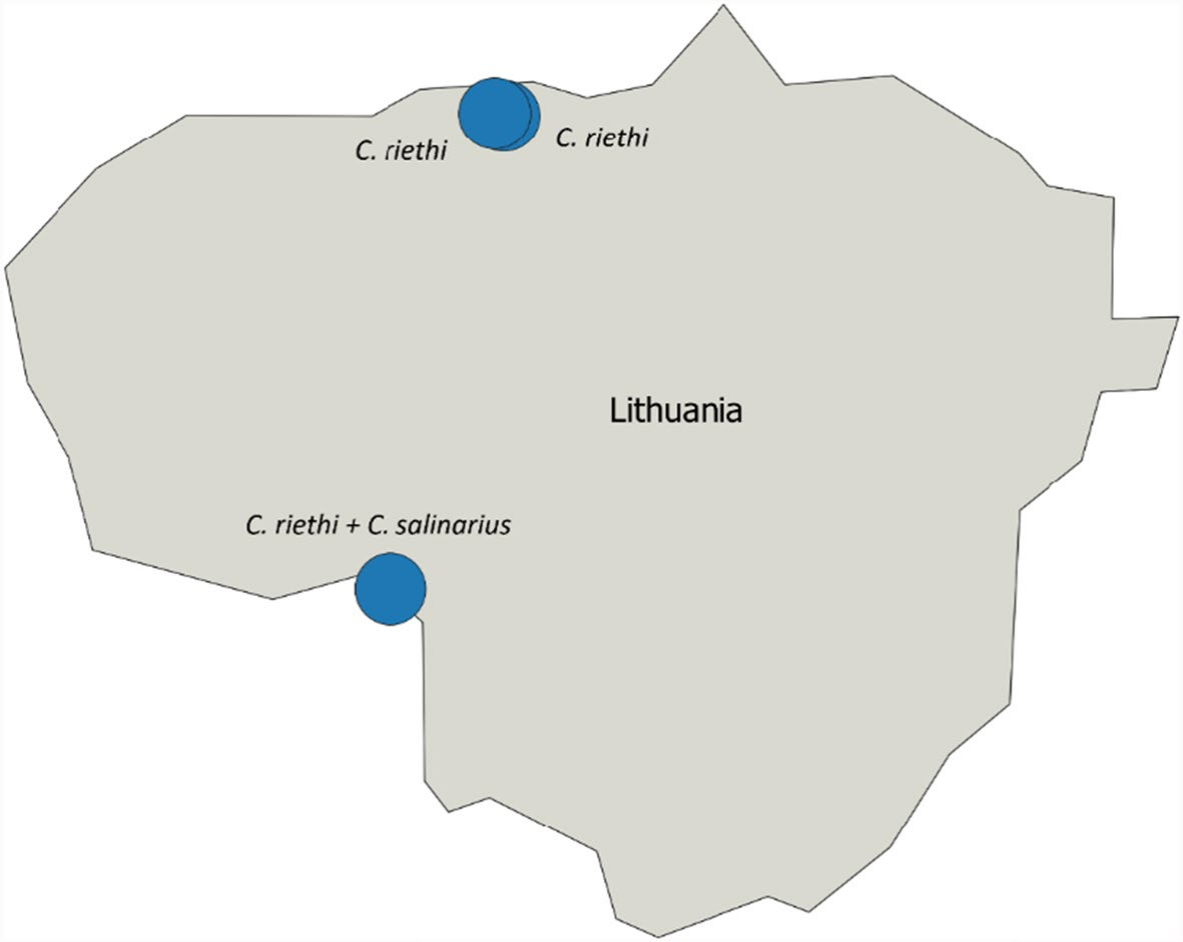
Location of *C. riethi* Kieffer and *C. salinarius* Kieffer, newly reported in Lithuania

*C. riethi* and *C. salinarius* [44] were morphologically identified for the first time in Lithuania using Mathieu et al. [36]. The identification of the species was confirmed using PCR-based methods for analysis of cytochrome c oxidase subunit I sequences (COI), which are used as barcoding for many insect species (Fig. 2).

### ASFV detection in insects collected on outbreak and control farms

**Fig. 3:**
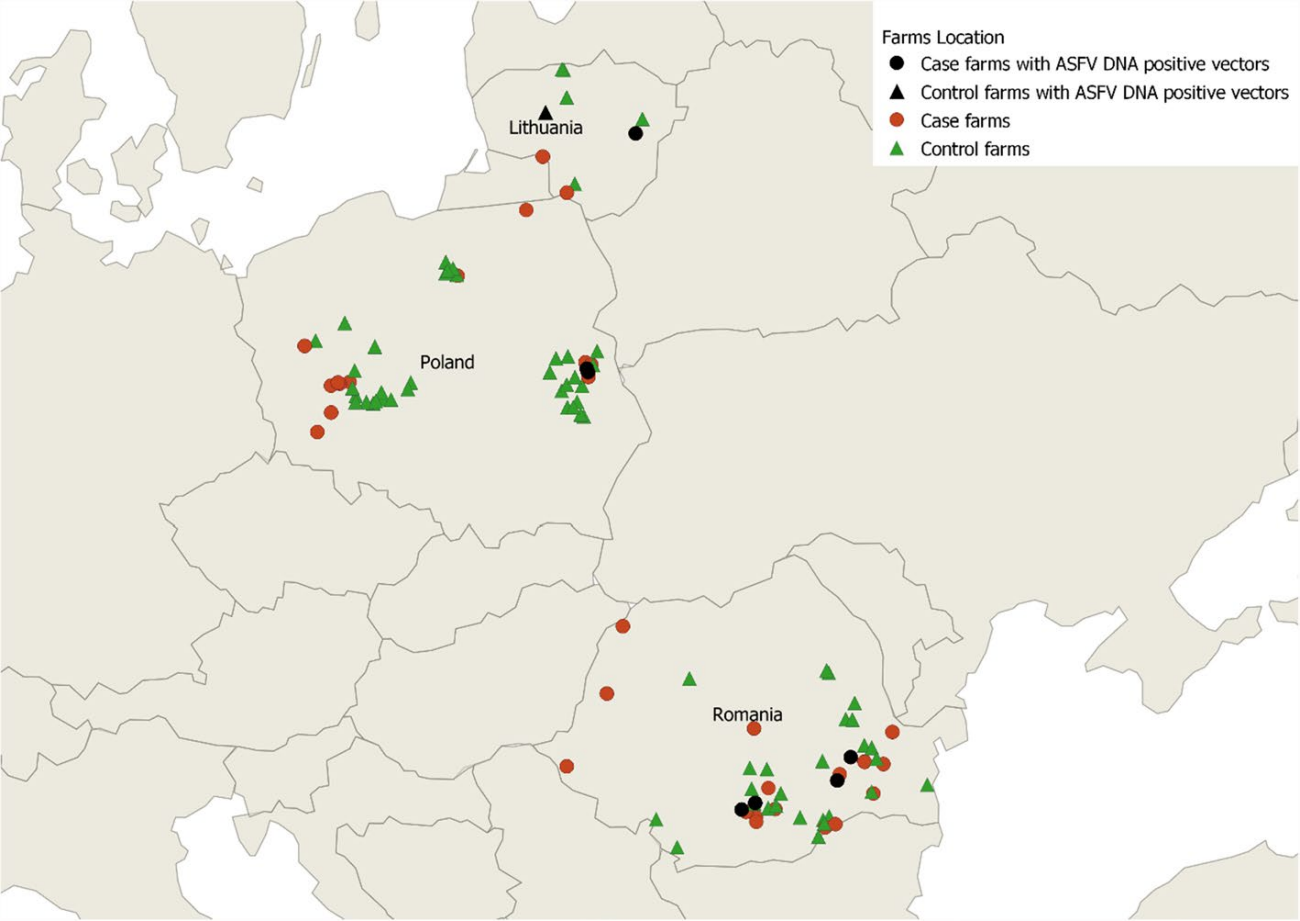
Locations of case and control farms in Lithuania, Poland and Romania where vector surveillance took place and ASFV-DNA-positive vectors were found

Vectors surveillance results were reported for 42 farms where ASF outbreaks were confirmed (case farms), and 70 control farms matched by farm size and location (Fig. 3). All case farms were visited within 2 days of ASF confirmation, and 85% of the control farms were visited within 2 weeks after the matching case farm’s outbreak confirmation, 8% between 2 and 3 weeks and 7% between 3 weeks and 1 month.

In Lithuania, three case and six control farms were included in the study, with ASFV-DNA-positive vectors detected on one case farm and one control farm (Fig. 3). In Poland, vector surveillance was conducted on 18 case farms and 36 control farms, with ASFV-DNA-positive vectors identified on 2 case farms. In Romania, vector surveillance was carried out on 21 case farms and 28 control farms, with ASFV-DNA-positive arthropods were found on 7 case farms (Fig. 3).

From the 1219 insect pools tested on the farms, 27 pools were positive for ASFV DNA. In 2021, 127 insect pools were tested for ASFV DNA, of which 18 insect pools were positive. In 2022, 368 insect pools were tested, with 1 pool positive for ASFV DNA. In 2023, 697 pools were tested, with 8 pools positive for ASFV DNA.

The detection of positive pools over tested pools in the 3 years together were spread over the months as following: two positives out of 8 tested pools in May, zero positive out of 331 tested in June, four positives out of 434 tested in July, 18 positive out of 285 tested in August, three positive out of 99 tested in September and zero positive out of 35 tested in October.

Across the three countries, a total of 8604 *Culicoides* spp. specimens were collected, of which 4962 were caught on control farms and 3642 were caught on case farms. Additionally, 742 *S. calcitrans* specimens were caught, of which 141 specimens on case farms and 616 on control farms (Table 1).

**Table 1:** Counts of *Culicoides* spp. and *S. calcitrans* pools and specimens per species collected from pig farms and the positive detections (in red) of ASFV DNA in pools and individual specimens

Species/genus	No. of pools	No. of specimens	Positive pools	No. of specimens in positive pools	No. of pools	No. of specimens	Positive pools	No. of specimens in positive pools
Farm category	Case farm				Control farm			
Lithuania								
*C. achrayi*	5	19	0	0	0	0	0	0
*C. circumscriptus*	2	5	0	0	3	5	0	0
*C. fascipennis*	3	7	0	0	4	11	0	0
*C. grisescens*	1	1	0	0	0	0	0	0
*C. impunctatus*	1	1	0	0	0	0	0	0
*C. kibunensis*	1	2	0	0	3	17	1	4
*C. nubeculosus*	2	5	0	0	0	0	0	0
*C. pallidicornis*	1	2	0	0	12	120	0	0
*C. pulicaris*	1	1	0	0	0	0	0	0
*C. punctatus*	74	697	1	10	55	472	0	0
*C. riethi*	4	16	0	0	6	6	0	0
*C. salinarius*	3	5	0	0	0	0	0	0
*Culicoides* spp.	0	0	0	0	3	6	0	0
*C. pictipennis*	0	0	0	0	1	2	0	0
*C. simulator*	0	0	0	0	3	3	0	0
Obsoletus complex^a^	20	121	0	0	19	165	0	0
Total Lithuania	118	882	1	10	109	807	1	4
Poland								
*C. achrayi*	13	102	0	0	16	94	0	0
*C. circumscriptus*	4	4	0	0	6	33	0	0
*C. fascipennis*	3	5	0	0	4	4	0	0
*C. pallidicornis*	29	252	0	0	70	649	0	0
*C. punctatus*	49	498	0	0	110	987	0	0
*C. riethi*	6	28	0	0	6	20	0	0
*C. furcillatus*	0	0	0	0	1	4	0	0
*C. grisescens*	0	0	0	0	1	1	0	0
*C. impunctatus*	0	0	0	0	2	2	0	0
*C. newsteadi*	0	0	0	0	1	1	0	0
*C. pulicaris*	0	0	0	0	3	41	0	0
Obsoletus complex^a^	60	361	0	0	167	1659	0	0
*Stomoxys calcitrans*	30	104	2	4	133	516	0	0
Total Poland	194	1354	2	4	520	4011	0	0
Romania								
*C. cataneii*	2	20	0	0	0	0	0	0
*C. circumscriptus*	1	10	0	0	1	10	0	0
*C. fagineus*	1	10	0	0	1	10	0	0
*C. fascipennis*	1	10	0	0	0	0	0	0
*C. longipennis*	1	10	0	0	0	0	0	0
*C. lupicaris*	4	40	1	10	0	0	0	0
*C. newsteadi*	47	470	5	50	14	140	0	0
*C. nubeculosus*	12	120	0	0	2	20	0	0
*C. punctatus*	79	790	17	170	37	370	0	0
*C. pulicaris*	0	0	0	0	3	30	0	0
*C. puncticollis*	0	0	0	0	2	20	0	0
Obsoletus complex^a^	3	30	0	0	6	60	0	0
*Stomoxys calcitrans*	11	22	0	0	50	100	0	0
Total Romania	162	1532	23	230	116	760	0	0
Total	474	3768	26	244	745	5578	1	4

^a^Includes *C*. *obsoletus* and *C*. *scoticus*

The most frequently caught *Culicoides* species across both farm categories were *C. punctatus* (*n* = 2657), Obsoletus complex (*n* = 2305), *C. pallidicornis* (*n* = 1023) and *C. newsteadi* (*n* = 631). The majority of *Culicoides* species positive for ASFV DNA belonged to *C. punctatus* (18 positive pools out of 410 tested) followed by *C. newsteadi* (5 positive pools out of 64 tested) and *Obsoletus* complex (7 positive pools out of 275 tested). None of the real-time-PCR-positive *Culicoides* pools had Ct values below 20 and were therefore not tested for virus isolation.

In Romania, pig DNA was identified in three pools of *C. punctatus*, three pools of *C. newsteadi* and one pool of *C. cataneii*. Two of these pools tested negative for ASFV DNA but with doubtful results, namely one of the *C. cataneii* pools with CT value of 37.3 and one of the *C. newsteadi* pools, with a Ct value of 36.2, but they were nonetheless tested for pig DNA, with negative results. All other pools that were negative for ASFV DNA were not tested for pig DNA. The other countries did not test for pig DNA in the arthropods.

Not all pigs on case farms are tested during culling, and unfortunately, information on estimated prevalence of ASF was not available during the surveys, but pigs were not yet culled on the seven farms where ASFV DNA-positive insect pools were detected. The number of pigs that were present on the case farms with positive insect pools were all of similar magnitude (between 1144 and 1788 pigs). Pig DNA was only found in samples from three of these seven case farms with positive ASFV DNA in the insect pools.

In total, two of the 224 *S. calcitrans* pools tested positive for ASFV DNA by real-time PCR. None of the PCR positive *S. calcitrans* pools yielded positive results in virus isolation.

Ct values of the positive pools, together with the results of the pig DNA analyses, are provided in Additional information 1. The detailed results of the vector surveillance have been published on the VectorNet data portal of the Global Biodiversity Information Facility (10.15468/rm3g5q).

### Statistical analysis

None of the performed statistical analysis identified any significant association of the investigated variables (sampling year, sampling month, presence of pigs on the farm during vector survey, numbers of insects in the pool, country, region, farm category (case or control) and the *Culicoides* species) with the presence of ASFV DNA in the *Culicoides* pools.

## Discussion

Surveillance of vectors was conducted on 42 outbreak farms and 70 control farms. ASFV-DNA-positive *Culicoides* pools were found on eight case farms and one control farm. Additionally, ASFV DNA positive *S. calcitrans* flies were found on two case farms. While there was a higher occurrence of DNA positive insect pools on case than control farms, statistical analysis (random Forest, classification tree and logistic regression statistical analysis) showed no significant association between farm category and ASFV DNA test results. This lack of significance is probably due to the small sample size because fewer outbreaks than anticipated occurred on commercial pig farms during the study period.

ASFV could not be isolated from any of these DNA positive insect pools. The inability to detect viable ASFV was likely due to the very small quantities of blood available in the *Culicoides* pools tested by real-time PCR. Such pools comprised half the volume of homogenates from up to 20 *Culicoides* specimens, or fewer when fewer individuals of a given species were caught. The mean blood volume of *Culicoides* have been estimated to range from 0.03 to 0.08 μL using spectrophotometry and 0.01 to 0.11 μL by weighing [45]. This is about 10 times smaller than blood meals of mosquito species [46], and 20 times smaller than blood meal volumes of *S. calcitrans* [47].

In addition, ASFV’s short survival time at higher summer temperatures may have contributed to the inability to recover viable virus.

ASFV survival has been studied in several matrices. At room temperature, it has been shown to survive up to 3 days in pig faeces and 5 days in urine [48], less than 1 day on cereal grains and up to 30 days on soy oil seeds and soybean meal [49, 50]. Specific studies investigating the survival of ASF in insects, such as the study of Blome et al. [51] observed that ASFV could be isolated from *Stomoxys calcitrans* flies kept in an incubator at 20 °C up to 2 days after feeding on viraemic blood. However, higher temperatures significantly reduced virus survival time. Although the traps in this study were placed in the shade, it is possible that high temperatures inside the traps were reached, negatively affecting the survival time of ASFV on mechanical vectors. Thus, while ASFV can be isolated from insects for short periods under laboratory conditions, environmental factors, particularly high summer temperatures, likely inhibit its long-term survival in insects during filed conditions.

Nonetheless, the CT values of the 25 positive *Culicoides* pools ranged from 25.33 to 34.93, with half of the positive pools with Ct values between 25.33 and 30.24, indicating higher viral DNA loads in these pools. Although the study focused on the potential mechanic transmission by biting midges of the genus *Culicoides*, the low Ct values in these samples could also support the hypothesis of transmission through blood feeding, given the high titres of ASFV in pig blood. However, if the reasoning regarding the viability of ASFV in *S. calcitrans* extends to *Culicoides*, and considering the time between blood meals in *Culicoides*, which are assumed to only feed once per gonotrophic cycle [52] approximately every 4 days at 25 °C, it is less likely that *Culicoides* act as vectors through blood feeding.

To increase the detectability of the virus, all individual insects were pooled and tested regardless of their sex or life stage. It was hypothesized that, during mechanical transmission, the virus may remain on the mouth parts, regardless of whether the females is parous, nulliparous or blood engorged. It should be acknowledged that distinguishing between unpigmented females, pigmented females, blood-fed females, and males would have been valuable. Future studies should incorporate this level of detail to enhance our understanding, particularly regarding ASFV detection in blood-fed versus non-blood-fed specimens.

The findings align with previous studies that demonstrated the presence of ASFV DNA in *Culicoides* [20] and *Stomoxys calcitrans* [20, 22], without isolating viable ASFV. They add to the growing body of evidence suggesting that *Culicoides* and *S. calcitrans* could serve as a mechanical vector of the virus. The repeated detection of ASFV DNA in these insects highlights the need for further studies to clarify their role in ASF transmission.

It is possible that *Culicoides* merely landed on or fed from contaminated materials on the farm, without actively contributing to the transmission of ASFV. The finding of the ASFV-DNA-positive biting midge on a control farm was not followed by infection of the pigs on the farm, as the farm remained ASF-free. However, this observation was limited to a single farm, and the biosecurity measures and insect control strategies in place may certainly have played a role in protecting the pigs from infection. The control farm was located 131 km from the nearest reported outbreaks in wild boar, suggesting unnoticed spread within the wild boar population. Given that the the typical fly range of biting midges does not exceed 1 km [24], it is unlikely that they played a role in transmitting the virus over such a long distance. Cross-contamination of samples was considered improbable, as separate traps and collection teams were used for case and control farms.

It should be noted that the specific species found positive on the control farm, i.e., *Culicoides kibunensis,*, is not well-documented with regard to its association with mammals. It is possibly a generalist feeder, or even primarily a bird-feeder [53].

Among the ASFV-DNA-positive arthropods caught on the pig farms, the majority belonged to the species *C. punctatus* (18 positive pools), followed by Obsoletus complex (*7* positive pools) and *C. newsteadi* (5 positive pools). These were also to the most frequently observed *Culicoides* species on the studied pig farms. In Romania, the only country where the insect pools were tested for host DNA, three *C. punctatus* pools, three *C. newsteadi* pools and one *C. catanei* pool tested positive for pig DNA, which confirms these ASFV-DNA-positive insects must have been in direct or indirect contact with pigs. It should be noted that evidence of insects feeding on pigs without acquiring of ASFV DNA would also have been of substantial value, as it could indicate that not all pigs on the farms were infected. Unfortunately, only ASFV-positive insects were tested for pig DNA. The number of infected pigs on the farms was not available, despite its potential influence on the probability of ASFV/DNA detection in the insects.

The fact that, from only three out of the seven case farms, where ASFV DNA was detected in 23 *Culicoides* pools, pig DNA was found, despite a similar number of pigs on the farms, is remarkable. Olesen et al. [9] mentioned that insect samples could be more sensitive to degradation than samples from mammals when detecting mitochondrial DNA. They suggested the use of next-generation sequencing to investigate the source blood meals from insects collected in the field.

The freeze–thaw cycle is unlikely to have affected the integrity of the insect samples for the detection of ASFV DNA, given the strong resilience of the virus under frozen conditions [54, 55]) and the fact that only a single thaw was required. However, it may have had an effect on the detection of pig DNA in the insect samples.

Tomazatos et al. [28] studied blood meals of common *Culicoides* species in the Danube Delta in Romania. The researchers identified *Sus scrofa* as the second common blood meal of *C. punctatus*, after cattle. The *S. scrofa* DNA in the blood meals identified in the biting midges of the genus *Culicoides* caught in the study of Tomazatos et al. was from feeding on wild boar, as there were no pig farms in the vicinity of the trap sites.

This study marks the first identification of *C. riethi* and *C. salinarius* in Lithuania. These vector species had not been previously recorded in the country, likely because earlier investigations were not performed near pig farms or habitats with similar suitable larval breeding sites. It was observed that *C. riethi* larvae develop in water bodies with extremely low pH and a high concentrations of sulphates, while larvae of *C. salinarius* can thrive in saline waters [56]. Our findings demonstrate the affinity of *Culicoides* to pig farms (8604 *Culicoides* spp. specimens were detected on 112 farms over one night). While the actual number of midges captured in a single night can vary widely, depending on local conditions, trap type and trap configurations, comparing this with numbers of *Culicoides* caught on cattle or horse farms for instance, average catches of 1438 and 1161 midges per week, respectively, were reported in Poland [57].

Notably, species such as *C. punctatus* and *C. newsteadi* were particularly prevalent on the pig farms in this study. The peak of positive pools was observed in August, with 6% of all the tested pools in August testing positive. The possible feeding of *Culicoides* on pigs increases the likelihood of virus acquisition and subsequent dissemination to other farms, provided the virus would remain infective in the insect. Furthermore, environmental factors, including farm management practices, the density of pigs and other hosts, and proximity to natural habitats may influence the abundance and feeding behaviour of *Culicoides*, further shaping their potential role in ASFV spread.

Additional determinants related to husbandry practices that may have influenced the occurrence of ASF on commercial farms were evaluated in this case–control study and reported by Dhollander et al*.*[27]. One notable finding was the significant difference in the use of insect nets between case and control farms, suggesting that insect nets could play a role in preventing the introduction of infected arthropods. Another interesting finding was that the application of manure from other farms near pig farms was identified as a risk factor for the occurrence of ASF. Manure may have served as an attractant for flies, thereby increasing the risk of disease transmission. In addition, various types of dung have been shown to serve as suitable breeding sites substrates for the development of *Culicoides* vector species [58, 59], highlighting the association between specific *Culicoides* species and manure as a breeding habitat.

## Conclusion

These findings emphasize the importance of implementing vector control strategies in ASF-affected areas. While the primary transmission routes of ASFV remain direct and indirect contact, such as through fomites, the potential role of incidental mechanic vectors such as *Culicoides* spp. or *S. calcitrans* should not be ignored. Future research should focus on elucidating the dynamics of ASFV infection in *Culicoides*, including the potential for virus survival in the mouth parts and gut of the biting midges of the genus *Culicoides*, and the conditions that may facilitate vector-borne transmission. Such insights are crucial for refining biosecurity measures and mitigating the risk of ASFV transmission risk in pig populations.

Vector importance varies by virus – some, such as the rinderpest virus, have negligible mechanical transmission, while others, such as lumpy skin disease virus, rely heavily on vectors for spread. Detecting a virus in blood-feeding insects does not necessarily confirm their role in transmission, as they may simply have fed on infected hosts without facilitating further spread. Since laboratory data alone are insufficient to establish the role of a vector in virus transmission, vector involvement is typically determined by linking disease patterns with vector distributions in the field.

## Data Availability

Ct values of positive samples were added in the Additional Material annexed to the manuscript. The vector surveillance data are published on the Global Biodiversity Information Facility: 10.15468/rm3g5q.

## Appendix 1

Ct values of ASFV-DNA-positive insect pools

**Table:**

Farm category	Species	Pool code	Country	Number of specimens in pool	Ct value	DNA of *Sus scrofa*
Case	*Culicoides lupicaris*	2_17	RO	10	30.24	Negative
Case	*Culicoides newsteadi*	2_7	RO	10	33.45	Negative
Case	*Culicoides newsteadi*	4_11	RO	10	25.6	Negative
Case	*Culicoides newsteadi*	4_17	RO	10	28.58	Negative
Case	*Culicoides newsteadi*	III_3	RO	10	33.17	Positive
Case	*Culicoides newsteadi*	VI_1	RO	10	32.79	Positive
Case	*Culicoides punctatus*	2_12	RO	10	32.76	Negative
Case	*Culicoides punctatus*	4_10	RO	10	28.8	Negative
Case	*Culicoides punctatus*	4_23	RO	10	32.46	Negative
Case	*Culicoides punctatus*	4_3	RO	10	28.76	Negative
Case	*Culicoides punctatus*	5_13	RO	10	33.58	Negative
Case	*Culicoides punctatus*	5_15	RO	10	30.87	Negative
Case	*Culicoides punctatus*	5_18	RO	10	30.07	Negative
Case	*Culicoides punctatus*	5_20	RO	10	27.24	Negative
Case	*Culicoides punctatus*	5_24	RO	10	25.33	Negative
Case	*Culicoides punctatus*	5_25	RO	10	28.09	Negative
Case	*Culicoides punctatus*	5_26	RO	10	26.72	Negative
Case	*Culicoides punctatus*	5_27	RO	10	31.57	Negative
Case	*Culicoides punctatus*	5_28	RO	10	26.89	Negative
Case	*Culicoides punctatus*	9_1	RO	10	32.67	Negative
Case	*Culicoides punctatus*	II_14	RO	10	31.36	Positive
Case	*Culicoides punctatus*	II_4	RO	10	29.39	Positive
Case	*Culicoides punctatus*	VI_3	RO	10	33.37	Positive
Case	*Culicoides punctatus s.l.*	69a	LT	10	34.93	Not tested
Case	*Stomoxys calcitrans*	26,720(13)	PL	2	32.05	Not tested
Case	*Stomoxys calcitrans*	26,721(12)	PL	2	34.21	Not tested
Control	*Culicoides kibunensis*	190a	LT	4	33.97	Not tested

LT, Lithuania; PL, Poland; RO, Romania
